# Good is up—spatial metaphors in action observation

**DOI:** 10.3389/fpsyg.2015.01605

**Published:** 2015-10-20

**Authors:** Janna M. Gottwald, Birgit Elsner, Olga Pollatos

**Affiliations:** ^1^Department of Psychology, Uppsala University, Uppsala, Sweden; ^2^Department of Psychology, University of Potsdam, Potsdam, Germany; ^3^Department of Health Psychology, Ulm University, Ulm, Germany

**Keywords:** embodied cognition, spatial metaphors, emotional valence, action observation, action perception

## Abstract

Positive objects or actions are associated with physical highness, whereas negative objects or actions are related to physical lowness. Previous research suggests that metaphorical connection (“good is up” or “bad is down”) between spatial experience and evaluation of objects is grounded in actual experience with the body. Prior studies investigated effects of spatial metaphors with respect to verticality of either static objects or self-performed actions. By presenting videos of object placements, the current three experiments combined vertically-located stimuli with observation of vertically-directed actions. As expected, participants’ ratings of emotionally-neutral objects were systematically influenced by the observed vertical positioning, that is, ratings were more positive for objects that were observed being placed up as compared to down. Moreover, effects were slightly more pronounced for “bad is down,” because only the observed downward, but not the upward, action led to different ratings as compared to a medium-positioned action. Last, some ratings were even affected by observing only the upward/downward action, without seeing the final vertical placement of the object. Thus, both, a combination of observing a vertically-directed action and seeing a vertically-located object, and observing a vertically-directed action alone, affected participants’ evaluation of emotional valence of the involved object. The present findings expand the relevance of spatial metaphors to action observation, thereby giving new impetus to embodied-cognition research.

## Introduction

The ups and downs of life. In everyday speech many metaphors link vertical space and emotional valence. People, objects, and actions with positive attributes are associated with metaphors pertaining to their physical highness, while those perceived negatively are often related to physical lowness. These fundamental concepts are thought to develop early in life—to some extent in the preverbal period (Lakoff and Johnson, 1980; Barsalou, 2009). Accordingly, metaphors are more than a linguistic phenomenon. From an embodied-cognition point of view metaphors are grounded in our bodies and therefore in our physical experience. In particular, spatial metaphors like “good is up” and “bad is down” are inseparable from their experiential basis and they enable us to orient within our world—they are the “heart of our conceptual system” (Lakoff and Johnson, 1999, p. 30; Glenberg and Kaschak, 2002; Casasanto, 2009; Shapiro, 2011).

Despite of the often-mentioned assumed embodied basis of spatial metaphors, much of the previous research on the relations between metaphors of vertical space and emotional valence has focused on the evaluation of static stimuli located within different vertical positions. One approach asked whether vertical position affects ratings of emotional valence for neutral stimuli. In their study on the connection between verticality and perceived physical attractiveness, Meier and Dionne (2009) presented several images of females and males, who scored similarly on attractiveness ratings, in various vertical positions. The expected results occurred only when participants saw images of their own sex: Participants rated the up-presented persons as more attractive than the down-presented persons. Similar results were found when participants rated up- versus down-presented images of persons in terms of higher versus lower religiousness as a positive value (Meier et al., 2007).

Another study examined whether vertical position influences (spatial) memory or attention for emotional stimuli. Crawford et al. (2006) found a typical bias, in that their participants remembered positive images from the international affective picture system (IAPS) as being located at higher screen positions, and negative images at lower positions, than they had been de facto. Likewise, in a study by Meier et al. (2007) on the association between vertical space and the divine, participants remembered “god”-like images metaphor-consistently as appearing higher, and “devil”-related images as appearing lower than neutral control pictures. In a categorization task, participants encoded “god”-related words (such as “Almighty”) faster if these were in a high vertical position as opposed to the low vertical position and “devil”-related words (such as “Lucifer”) faster if these were in a low vertical position as opposed to the high vertical position. These findings corroborate with a previous study in which Meier and Robinson (2004) let their participants evaluate affective words as either positive or negative. They found that the ratings were faster if the words were presented metaphor-consistently in vertical space.

While considerable body of work has demonstrated relations between vertical position and emotional valence, few studies have investigated the effect of metaphors by allowing individuals to act in the vertical dimension of space. For instance, Casasanto and Dijkstra (2010) studied the interaction between emotional memory and motion: While moving their hands up or down (by moving marbles), participants should tell a pleasant or an unpleasant past episode of their life. Participants were faster at retrieving memories if their movements and memory valence were congruent (e.g., moving marbles up and telling a positive episode), and they were also more likely to retrieve a memory whose valence was congruent with their movement.

Seno et al. (2013) built on these results and made use of illusory self-motion to further distinguish between effects of motion perception concerning the observer and concerning the observed stimulus. Actually stationary observers were presented with upward- or downward-moving patterns (inducing the reversed self-motion experience), and were asked to retrieve autobiographical episodic memories. Participants more likely recollected positive or negative memories in congruency with their illusory upward or downward self-motion direction, i.e., incongruent with the motion direction of the pattern. Thus, it is one’s perceived motion direction and not the actual motion direction of the stimulus that drives the mood-congruency effect, which highlights the importance of the body in metaphor-related effects.

All previous studies have investigated the relationship between metaphors of vertical space and emotional valence either by having participants look at objects located in vertical space, or allowing them to place the objects themselves. Combining these aspects is an interesting approach, especially because we learn various motoric behaviors and gain many of our experiences while observing others interacting with objects (Mattar and Gribble, 2005; Elsner, 2009). The present research sought to investigate whether observing a person acting on an object in vertical space can also trigger the metaphor “good is up” (respectively “bad is down”) by presenting the objects in the context of dynamic actions mimicking real-life experiences. Specifically, the current work asked whether evaluations of emotional valence of everyday objects are more positive when the objects are observed being placed up (i.e., higher in vertical space) as compared to when they are observed being placed down (i.e., lower in vertical space).

## Study 1

In an initial study, we used two vertical positions (“up” and “down”). We based our hypotheses on effects of the vertical positions and expected that emotionally neutral objects would be rated more positively in terms of emotional valence when they are observed being placed up in vertical space as compared to when they are observed being placed down. It is important to note that we expected effects of vertical positioning only for ratings that involve emotional valence (e.g., gift likelihood, willing to pay), but not for ratings related to the cover story, i.e., ratings aimed at object physicality (i.e., attractiveness of color or shape).

### Method

#### Participants

The sample consisted of 41 German university students (age *M* = 22.71, SD = 2.75; all female and right-handers) who received a course credit for participation. Participants were recruited with announcement advertising a study on product evaluation. None of the participants was aware of the purpose of the experiment. Approval from the local ethical boards was obtained in accordance to the Declaration of Helsinki.

#### Stimuli

**FIGURE 1:**
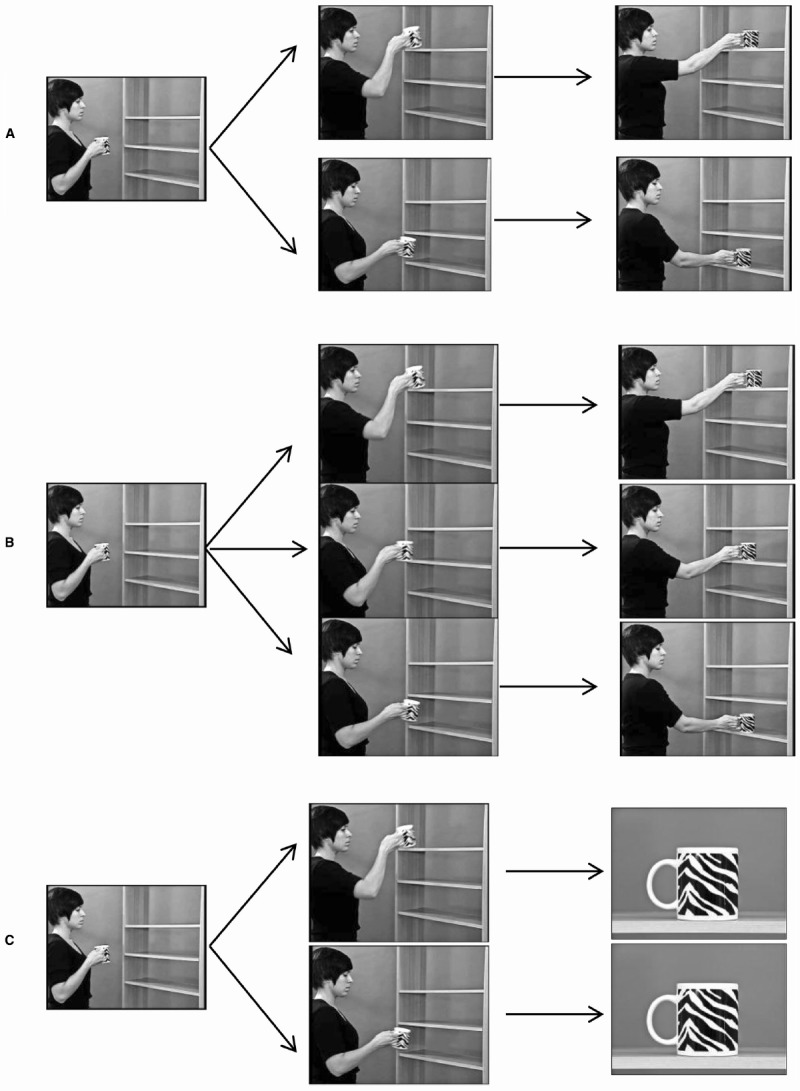
**Screen shots of the videos used in Study 1 (A), Study 2 (B), and Study 3 (C).** Starting position (left), end of the upward/downward movement (middle), and end positions of the placements (right; Study 1: up, down; Study 2: up, middle, down; Study 3: full-screen picture of the cup, spatially neutral position).

Videos showing the placement of an emotionally neutral object in vertical space served as stimuli: A woman placed a cup in a wooden bookcase with three vertically positioned shelves (Figure 1A). The woman almost filled the left half of the screen, and the bookcase filled the right half. The positions of the shelves were defined in relation to the woman’s body. The lowest shelf (“down”) was on the same height as her elbow, when her arm was to her side. The “middle” shelf was the same height as her chest, while the highest shelf (“up”) was the same height as her chin. In order to create similar conditions with regard to motor effort, the distances between the shelves were of equal size, and they were determined in relation to the length of the woman’s forearm.

Twenty-four cups, differing in form, color, design, and two placements (“up” and “down”) were used to create 48 video stimuli. Every cup was shown in each position to rule out the influence of possible preferences for certain cups. Each video took 6 s and started with showing the woman in profile holding a cup in her right hand, in height of the middle position. The woman looked first at the bookcase and then at the cup in her hand. She then moved her right arm and placed the cup on one of the shelves (“up” or “down”) and held the cup for the last 2 s of the scene. Her movement was such that she did not obstruct the cup’s visibility to the observer with her hand or her arm.

#### Procedure

Participants were seated in an experimental cabin and watched the videos. They first saw two training trials, using videos with a cup that was not in the final sample. Then, the 48 stimulus videos were shown on an 18-inch screen to every participant in a randomized order. Prior to the presentation of each video, a 50 Hz tone and a centered fixation cross were presented. After each video, six items demanding ratings of different aspects of the presented cup, including two main target variables of emotional valence, were presented each after another on the screen (i.e., 288 ratings in total).

The first three items were aimed at object physicality underlining the cover story that this study was about product evaluation. Item 1 was about shape (“How attractive do you find the shape of the cup?”) and item 2 was about color (“How attractive do you find the color of the cup?,” English equivalents). These two items were later combined into one mean rating of Esthetics. Item 3 (“How do you appraise the usability of the cup?”) was not evaluated. The next two items were target items of emotional valence being associated with the vertical placement of the object: “How likely is it that you would choose this cup as a gift for your best female friend?” (item 4: Gift likelihood) and “How much would you pay for this cup?” (item 5: Willing to pay; cf. Lee and Schwarz, 2010). Item 6 asked explicitly about emotional valence: “How much do you like this cup?” (Liking).

Below each question, a horizontal visual analog scale was presented. Scales ends ranged from 1 (items 1, 2, 3, 4, 6: *not at all*; item 5: *0.99€*) to 10 (items 1, 2, 3, 4, 6: *very much*; item 5: *4.59€*). For item 5, amounts of money were specified, increasing by 0.40 € across each of the 10 steps. Participants answered by pressing a key on a German keyboard and were instructed to answer as quickly and as accurately as possible. The middle line of letter keys was used, from “A” = “1” to “Ö” = “10”. After the experiment, participants were asked about the assumed purpose of the experiment. Presented with two options “product evaluation” and “other” (that had to be defined by a free answer), they could either tick one or both options. The experiment took about 30 min in total.

#### Data Analysis

The average ratings for every cup in each spatial position were calculated and averaged across all cups. Then, separate analyses of variance (ANOVAs) for the four aspects (i.e., Esthetics, Gift likelihood, Willing to pay, Liking) were conducted with the within-subjects factor spatial position (up, down).

### Results

**FIGURE 2:**
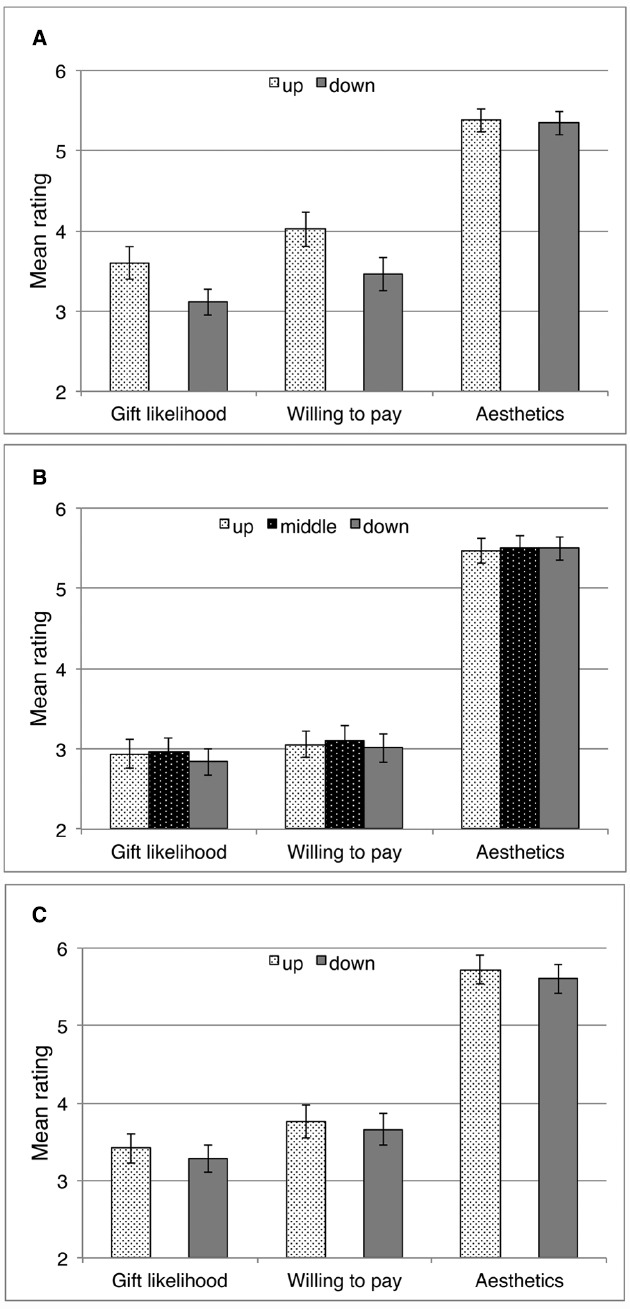
**Mean ratings of Gift likelihood (choosing the cup as a gift for a friend), Willing to pay (amount of money), and Esthetics (attractiveness of the cup’s shape or color) as a function of the cups’ vertical placement “up” versus “down” in Study 1 (A), Study 2 (B), and Study 3 (C), as well as “middle” in Study 2 (B).** Error bars indicate standard errors. Scale: 1 = *not at all/0.99*€, 10 = *very much/4.59*€.

Testing of normal distribution of the data was performed using Shapiro–Wilk tests and revealed no violation of normality. There was a significant main effect of spatial position for Gift likelihood, *F*(1,40) = 6.48, *p* = 0.015. As hypothesized, Gift likelihood was higher for objects observed to be placed up than for objects observed to be placed down, MD (mean difference) = 0.48, SD = 0.19, *r* = 0.14. Hence, cups that were placed on the higher shelf were more likely to be chosen as possible gifts than were cups placed on the lower shelf (see Figure 2A).

The main effect of spatial position for Willing to pay was also significant, *F*(1,40) = 5.52, *p* = 0.024. As expected, the indicated amount of money was higher for objects observed to be placed up as compared to down, MD = 0.56, SD = 0.34, *r* = 0.12. Thus, participants would have paid more for cups that were placed on the upper shelf than for cups placed on the lower shelf.

There also was a significant main effect of spatial position on the item asking explicitly about Liking, *F*(1,40) = 8.21, *p* = 0.007. Participants liked the cups that were observed to be placed up more (*M* = 4.78; SD = 1.68) than those that were observed to be placed down (*M* = 3.98; SD = 1.19), MD = 0.81, SD = 0.30, *r* = 0.17.

As expected, there was no significant main effect of spatial position for Esthetics (mean scores across items 1 and 2), *F*(1,40) = 0.32, *p* = 0.572. Ratings concerning the cup’s shape or color did not differ significantly for the observed vertical placements, MD = 0.44, SD = 0.57, *r* < 0.01.

It has to be noted that the reported mean ratings were in a rather low range compared to possible scores up to 10 (see Figure 2A). The differences between ratings across the conditions and the effect sizes were small (cf. Cohen, 1992).

The systematic question about the assumed purpose of the experiment revealed that none of the participants was aware of the purpose. Most participants believed the study was only about “product evaluation” (90.24%); few thought it was about “product evaluation” and “drinking behavior” respectively “thirst” (7.32%); one participant believed the study was only about “thirst” (2.44%).

### Discussion

The present study tested the assumption that observation of a vertically-directed action affects the evaluation of emotional valence of an object via influence of the spatial metaphors “good is up” or “bad is down.” Indeed, main results show that observing a placement of an emotionally neutral object leads to a more positive rating of emotional valence when the object is placed up as compared to down.

Our hypotheses were supported by the results. As expected, we did not find any differences between the vertical positions in participants’ ratings for the two cover-story items about object physicality (i.e., the object’s shape or color). However, vertical position had the expected significant effects on all three target items of emotional valence. Although it has to be noted that the effects were small, participants rather chose a cup as a gift for a friend, were willing to pay more, and explicitly liked the cup better when this cup was observed being placed up as compared to being placed down. Hence, measuring emotional valence directly did not minimize the effects, but revealed similar effects compared to more indirect measurements (as Gift likelihood or Willing to pay). In sum, Study 1 extended the results of previous research on the effects of spatial metaphors on ratings of emotional valence (e.g., Meier et al., 2007; Meier and Dionne, 2009) to observed vertically-directed actions and vertically-located end positions of objects.

## Study 2

Given the small, but significant effects of Study 1, we aimed at replicating our findings and at strengthening our initial interpretation in Study 2. Moreover, to investigate whether an additional differentiation in vertical space interacts with emotional valence and to examine the specific effects of vertical placement, we used three vertical positions (up, middle, down). This was done because just comparing “up” versus “down” does not inform about whether each positioning actually changes emotional valence compared to a neutral condition (i.e., middle). Because we did not find different effects of vertical position on explicit or implicit ratings of emotional values in Study 1, we used only the two more implicit items of emotional valence in Study 2 (i.e., Gift likeliness, Willing to pay). Again, we expected first, that emotional valence of the cups would be rated more positively when cups are observed being placed up, as compared to down, in vertical space. Second, we expected that cups observed being placed “up” (spatially higher) should be rated more positively than those being placed in the “middle” position. Third, we hypothesized that cups observed being placed “down” (spatially lower) should be rated less positively than those being placed “middle.” As in Study 1, we expected effects of vertical positioning only for ratings that involve emotional valence (i.e., Gift likeliness, Willing to pay), but not for ratings related to the cover story (i.e., Esthetics).

### Method

#### Participants

The sample consisted of 48 German university students (age *M* = 24.85 years, SD = 5.86; eight male, all right-handers) who received a course credit for participation. Participants were recruited with announcement advertising a study on product evaluation. None of the participants was aware of the purpose of the experiment. Approval from the local ethical boards was obtained in accordance to the Declaration of Helsinki.

#### Stimuli

The videos of Study 1 and 24 additional videos showing the placement of the same cups on the “middle” shelf served as stimuli, resulting in 72 video stimuli (Figure 1B).

#### Procedure

The procedure was the same as in Study 1, except for the number of trials and items: Participants first saw three training trials and then 72 stimulus videos. After each video, items 1 to 5 from Study 1 were shown, thereby omitting the rating of explicit Liking (i.e., 360 ratings in total). The experiment took about 30 min in total.

#### Data Analysis

Data analysis was identical to that in Study 1, except for conducting separate ANOVAs for only three aspects (i.e., Esthetics, Gift likelihood, Willing to pay) and with three stages of the within-subjects factor spatial position (up, middle, down).

### Results

Shapiro-Wilk tests revealed no violation of normal distribution of the data. We were able to replicate the results of Study 1. There was a significant main effect of spatial position for Gift likelihood, *F*(2,94) = 4.86, *p* = 0.010 (Figure 2B). In line with our hypotheses, pairwise comparisons (Bonferroni corrected) demonstrated that Gift likelihood was higher for objects observed to be placed “up” as compared to “down,” MD = 0.10, SD = 0.04, *p* = 0.014, *r* = 0.14, and also was higher for “middle” than for “down,” MD = 0.11, SD = 0.04, *p* = 0.009, *r* = 0.15. However, the difference between “up” and “middle” was not significant, MD = 0.02, SD = 0.04, *p* > 0.999, *r* < 0.01. Hence, cups that were placed higher in vertical space (i.e., “up” or “middle”) were more likely to be chosen as possible gifts than cups placed on the lowest shelf (“down”), but placing the cup “up” as compared to “middle” did not have a specific influence on Gift likelihood.

The main effect of spatial position for Willing to pay was also significant, *F*(2,94) = 3.14, *p* = 0.048. As hypothesized, pairwise comparisons (Bonferroni corrected) revealed a significantly higher rating for objects observed to be placed “middle” as compared to “down,” MD = 0.10, SD = 0.04, *p* = 0.022, *r* = 0.12. However, the differences between “up” and “middle,” MD = 0.06, SD = 0.04, *p* = 0.654, *r* = 0.03, or between “up” and “down,” MD = 0.05, SD = 0.04, *p* = 0.666, *r* = 0.03. Thus, participants would have paid more for cups that were placed on the middle shelf than for cups placed on the lowest shelf, but again, there were no specific relative effects of placing the cups on the highest shelf.

As expected, there was no significant main effect of spatial position for Esthetics (mean scores across items 1 and 2), *F*(2,94) = 0.54, *p* = 0.584. Ratings of the cup’s shape or color did not differ significantly for the observed placements.

There were only few male participants, but analyzing only the female participants yielded the same results. Therefore, we decided to keep all participants for the sake of increasing test power. As in Study 1, the mean ratings in Study 2 were in a rather low range, and the reported effect sizes were small according to Cohen (1992).

### Discussion

Study 2 confirmed that observation of a vertically-directed action affects the evaluation of emotional valence of an object via influence of spatial metaphors.

In general, our hypotheses were supported by the results. As expected, we again did not find any significant difference of spatial positioning in the cover-story ratings (i.e., attractiveness of the cup’s shape or color). For the ratings of emotional valence, our first hypothesis that observing the cups being placed “up” would lead to more positive ratings as compared to “down” was partly confirmed (only for Willing to pay). Again, all significant effects were small. Our third hypothesis that observing the cups being placed “down” would lead to more negative ratings as compared to “middle” was fully confirmed (for Gift likelihood and Willing to pay). However, there was no support for our second hypothesis that observing the cups being placed “up” would lead to more positive ratings as compared to “middle.”

It is interesting to note that “bad is down” seemed to have larger impact on participants’ ratings as compared to “good is up.” In demonstrating a specific relative effect for “down/negative,” but not for “up/positive” as compared to “medium/neutral,” the findings of Study 2 relate our research to previous work on the so-called negativity bias in the processing of emotional information. There is ample evidence that emotionally negative information is processed faster and has larger effects on the perceiver’s behavior than has emotionally positive information (e.g., see Cacioppo and Gardner, 1999, for review). However, further research is needed to study the specific effects of spatial metaphors like “good is up” or “bad is down” on participants’ ratings of emotional valence.

## Study 3

The results of Studies 1 and 2 provoke another interesting question, namely which part of the observed action triggered the spatial metaphor: the vertically-directed movement or the vertically-located end position of the object? In order to distinguish between the effects of those two parts of the action, we presented modified videos in Study 3, showing the cup being moved upward or downward, respectively, but we removed the final 2 s in which the cup was placed on the (up, middle, down) shelf and was held motionless. Instead, we presented a full-screen picture of the single cup, without any spatial reference. If the observation of the vertically-directed movement is crucial for the effect of spatial metaphors on the evaluation of the involved object’s emotional valence, we should obtain the same effects as in Studies 1 and 2. In contrast, if it is the static vertically-located end position that drives the effect, there should be no differences in the ratings for the upward or downward movement in Study 3. Because of the assumed embodied basis of spatial metaphors (e.g., Lakoff and Johnson, 1999), we expected action observation to be relevant in Study 3. Hence, the cups should be rated more positively in terms of emotional valence when they are observed being moved upward as compared to downward. Similar to Studies 1 and 2, we expected effects of vertical positioning only for ratings that involve emotional valence, but not for ratings of Esthetics.

### Method

#### Participants

The final sample consisted of 40 German university students (age *M* = 24.02 years, SD = 4.51; all female, all right-handers) who received either a course credit or 5 Euro for participation. Because of a technical problem, one additional participant had to be excluded from the sample. Participants were recruited with announcement advertising a study on product evaluation. Approval from the local ethical boards was obtained in accordance to the Declaration of Helsinki.

#### Stimuli

We modified videos of Study 1, showing the placement of the cups on two positions (“up” and “down”), resulting in 48 video stimuli (Figure 1C). The videos were cut before the cups reached their final position on the particular shelf. Thus, the upward respectively downward arm movement of the woman stopped just before the placing movement, at a natural turning point. Then a black screen was shown for 1 s, to make the transition less abrupt. Afterward, a full-screen picture of the same cup on a shelf (front view) was shown for 2 s. Because the woman and the bookcase were no longer visible, there was no spatial reference, so that the cup was presented at bigger size and in a neutral spatial position. The duration of presentation for the movement and for the cup in its static position was identical to that of Studies 1 and 2.

#### Procedure and Data Analysis

The procedure and data analysis were identical to that of Study 1, except for omission of the explicit item Liking. The experiment took about 30 min in total.

### Results

Shapiro–Wilk tests revealed no violation of normal distribution of the data. There was a significant main effect of spatial positioning for Gift likelihood, *F*(1,39) = 5.71, *p* = 0.022 (Figure 2C). As hypothesized, Gift likelihood was higher for objects observed to be moved upward than for objects observed to be moved downward, MD = 0.13, SD = 0.26, *r* = 0.13. Hence, presenting only the vertically directed movement, without the vertically-located end position, had the expected impact on participants’ ratings of how likely they would choose this cup as a gift for a friend.

However, the main effect of spatial position for Willing to pay was not significant, *F*(1,39) = 2.68, *p* = 0.110, although, as expected, Willing to pay was higher for objects observed to be moved upward as compared to downward, MD = 0.10, SD = 0.29, *r* = 0.06. Thus, the effect of observing only the vertically-directed movement apparently was not strong enough for inducing a significant difference in the amount of money participants were willing to pay for the objects.

Contrary to our hypothesis, the main effect of spatial position for Esthetics (mean scores across items 1 and 2) was also significant, *F*(1,39) = 4.28, *p* = 0.045. Yet, in line with the assumed impact of spatial metaphors, the attractiveness of the cup’s shape and/or color was rated higher for objects observed to be moved upward as compared to downward, MD = 0.12, SD = 0.26, *r* = 0.10.

Just like in Studies 1 and 2, the reported mean ratings were in a rather low range, and the reported effect sizes were small (Cohen, 1992).

### Discussion

Study 3 asked whether the observed effect of spatial metaphors on ratings of the emotional valence of objects also holds when only vertically-directed movements is shown, but the vertically-located end positions of the objects is removed and replaced by a full-screen picture of the object in a spatially neutral position. In general, the results partly confirmed our hypothesis that ratings in terms of emotional valence would be more positive when the object is observed to be moved upward as compared to downward. This was only confirmed for Gift likelihood, but not for Willing to pay. Moreover, against our hypothesis, presenting the vertically-directed movement and then the full-screen picture of the object also influenced participants’ ratings of Esthetics. Yet, the findings are in line with the assumed impact of spatial metaphors, in that the object’s shape or color was rated as being more attractive when the object was observed to be moved upward as compared to downward.

The significant effect of vertical movement on participants’ ratings of Esthetics was the main difference to the findings of Studies 1 and 2. The two items summed up here served as control for the cover story and did not differ depending on vertical space in the two previous studies. It can be speculated that presenting each object full-screen after having shown the vertically-directed movement directed participants’ attention to the physical features of the object, thereby increasing the subjective relevance of the cover-story related items 1 and 2. Yet, it is important to note that the ratings of the object’s attractiveness were in line with the expected effects of the presented vertical movements, that is, ratings were more positive for objects observed to be moved upward as compared to downward.

The effects of vertical positioning on Gift likelihood were the most stable over the three studies—the ratings for “up” were significantly more positive than for “down”—whereas the effects on Willing to pay were less pronounced—the difference for “up” and “down” was significant in Study 1, but not in Study 2, where only the difference for “middle” and “down” reached significance, and not in Study 3, although the non-significant difference was in the expected direction. It is up to further research to explore potential differential effects of spatial metaphors on various variables measuring indirect ratings of emotional valence.

In sum, even after removing the vertically-located end positions, there were differences in Gift likelihood, which suggests that observation of upward versus downward movements alone has an effect on ratings of the involved objects’ emotional valence. However, in Study 3, the differences between “up” and “down” were smaller than in Studies 1 and 2, and were only significant for one of the target variables, indicating that the combination of both—the vertically-directed movement and the vertically-located final position—may trigger spatial metaphors more strongly than does only the observation of the movement alone.

## General Discussion

Across three studies, the present findings demonstrate that observing somebody perform vertically-directed actions on an object influences ratings of the emotional valence of this object: objects observed to be placed in a higher position or to be moved upward were rated more positively with respect to likelihood to be chosen as a gift or to the amount of money one is willing to pay than were objects placed in a lower vertical position or moved downward. Spatial metaphors like “good is up” or “bad is down” thus not only work for static positions of objects or for self-performed actions. To our knowledge, this work is the first in demonstrating this effect for observed object-related actions.

Focusing on the motion aspect, our paradigm is an interesting expansion of studies demonstrating that static images of persons are rated more positively when being presented higher as compared to lower in vertical space (Meier et al., 2007; Meier and Dionne, 2009). In terms of an embodied-cognition approach, Meier et al. (2007) presented the outcome of positioning an object, whereas the present study depicted the act of positioning itself. If metaphors are grounded in physical experience (Lakoff and Johnson, 1999), the relation between vertical space and emotional valence should be triggered by observing a dynamic action, and this is supported by the present findings.

By using action observation, the previous studies also expand the previous findings on emotional valence and self-performed actions (Casasanto and Dijkstra, 2010). Our results indicate that seeing an object being placed up or down triggers the spatial metaphor “good is up” (respectively “bad is down”) in a similar way as does the actual performance of an upward or downward movement. This interpretation is in line with current research on the link between action production and action observation in human infants and adults (e.g., Rizzolatti et al., 2001; Falck-Ytter et al., 2006; Nyström et al., 2010). Perception and action are closely related and grounded in our bodies, as stated in theories of embodied cognition (Barsalou, 1999; Shapiro, 2011). In line with the idea that we gain experiences with objects by observing somebody acting with them (Elsner, 2009), we presented objects being moved rather than static objects, to enact “good is up” by observing objects being placed up or down. This captures potential effects of spatial metaphors with an increased external validity, because everyday life includes actions and moved objects.

In Studies 2 and 3, we largely replicated the results of Study 1. This replication allowed us greater confidence in the found effects, while addressing Lakens’ (2014) concern regarding the possibility of a Type 1 error and lack of replicability in (social) embodiment studies. The small, but significant effects in the current experiments ranged from *r* = 0.10 to *r* = 0.16 and are comparable to the small effect sizes of studies that have used static vertical positions (Meier et al., 2007: *r* = 0.28; Meier and Dionne, 2009: *r* = 0.08). However, Study 1 yielded more pronounced effects than did Study 2. In Study 1, the differences between the ratings for the two positions were greater, and every target comparison between up and down was significant. The use of the middle position in Study 2 did not lead to further differentiation, but rather seemed to suppress the effects of “good is up.” We introduced this additional position in vertical space (“middle”) to examine whether and how exactly vertical positioning interacts with emotional valence. Our findings indicate that participants perceived the middle shelf as the upper one compared to the “down” shelf, and that there was no further differentiation between “middle” (or rather “up”) and “up” (respectively “upper”). In fact, this interpretation may have been triggered by the specific relations between the acting woman’s body and the arrangement of the shelves in our study: the middle location (instead of the starting location of the object) could be interpreted as “up” relative to the location of the elbow of the acting person. The acting woman’s forearm is parallel to the ground in the “down” condition, but there is an angle between the ground and the forearm in the other two conditions. Thus, the similar results for the “up” and “middle” placements in Study 2 would strengthen the argument that spatial metaphors of emotional valence are rooted in bodily experience (Lakoff and Johnson, 1999; Casasanto, 2009).

An alternative explanation could be that putting cups on the middle shelf leads to positive ratings because of perceived low motor effort. Humans often prefer actions that involve less effort (Kurniawan et al., 2010). The most convenient way to place a cup in a bookcase would be to put it in height of the center of the body, because this movement does not require to raise or to lower the forearm. If the convenient way was the preferred one, observing “middle” placements could lead to a higher emotional valence of the involved object as compared to the less convenient “up” or “down” placements. However, this explanation can be ruled out in the present study. First, the shelves were relatively close to each other, so the required motor effort did not differ relevantly. Second, the middle shelf was not corresponding in height to the acting woman’s body center, but to her chest. In fact, the lower shelf was in height of her body center, and nevertheless, the ratings for the cups observed to be placed downward were the least positive.

With Study 3, we sought to disentangle which part of the observed action triggered the spatial metaphor: the vertically-directed movement or the vertically-located final position of the object? When presenting only the upward/downward movements, but substituting the vertically-located end points by full-screen pictures of the objects in a neutral spatial position, we replicated the results of Studies 1 and 2 for one of the target items indicating emotional valence of the objects (Gift likelihood), but not for the second target item (Willing to pay). Yet, Study 3 revealed an effect of the vertically-directed movement on participants’ ratings of the attractiveness of the object’s shape or color. These items served as control variables to increase the credibility of the cover story that this was a study on object evaluation. Overall, after removing the vertical end positions, observation of vertically-directed movements still affected participants’ ratings of the objects, in line with the expected effects of spatial metaphors. Admittedly, the observed condition differences in Study 3 were smaller than in Studies 1 and 2, which might be due to lower ecological validity (goal-directed movements usually have an end point in vertical space) or on the fact that we used only one part of the action (up-/downward movement) instead of both parts (movement and end position). A convincing argument would be that the spatial metaphors exert stronger effects when vertically-directed movements and vertically-located end positions are observed in combination, as compared to when either movement or end position are observed alone.

Additionally, it remains to explain why there was a significant difference between vertical positioning for the control items on attractiveness of object properties in Study 3, but not in the other two studies. This could be related to the more explicit presentation of the objects in Study 3. First, in the full-screen pictures, the cups were presented on their own without the women or the bookcase being visible, and second, the cups were presented at bigger size. The different presentation of the objects in their final position could be the reason why object properties and the ratings of attractiveness of the cups could have played a larger role in Study 3 than as compared to the first two studies. It would be interesting to investigate the effects of different object presentations (with and without human involvement) on participants’ ratings, but this was beyond the scope of this work.

The main limitations of the present study are the small effects and the difficulty of disentangling the effects of the vertically-directed movement or the vertically-located end position in Studies 1 and 2. However, as reported above, other studies on the impact of spatial metaphors also reported small effects (e.g., Meier et al., 2007; Meier and Dionne, 2009), and we were able to replicate the main effects of vertical positioning on the ratings of emotional valence across three experiments (Lakens, 2014). Moreover, in Study 3, we demonstrated that observing only the vertically-directed movement, without the vertical end position, significantly affected at least one of the target variables in the expected direction. We take this as evidence that action observation also contributed to the effects of Studies 1 and 2.

We conclude that emotionally neutral objects are rated more positively when they are observed to be placed upward (versus downward) in vertical space. This study is the first to find effects of vertical position on emotional appraisal of objects in action observation, and it therefore extends previous research showing that spatial metaphors like “good is up” or “bad is down” are triggered by seeing static objects at different vertical positions (e.g., Meier and Robinson, 2004; Crawford et al., 2006; Meier et al., 2007; Meier and Dionne, 2009) or by own execution of vertically-oriented actions (Casasanto and Dijkstra, 2010). We argue further that action observation plays an important role in activating spatial metaphors, as we were able to show that observation of vertically directed movements alone, without seeing the vertically-located end positions, influenced the emotional appraisal of the objects in the expected direction.

An interesting expansion of this work would be to investigate the specific effect of observation of human action, for instance by comparing it with observation of robotic action or self-propelled motion of inanimate objects. If spatial metaphors are grounded in our bodies (e.g., Casasanto and Dijkstra, 2010; Shapiro, 2011), they should only be triggered by observation of human actions (see also Falck-Ytter et al., 2006). Moreover, it would be interesting to study the assumption that embodied metaphors like “good is up” develop early in life (Lakoff and Johnson, 1999). The paradigm presented here could be used to investigate the effects of “good is up” already in infants or young children, for instance by examining looking or choice preferences for objects that have been observed being placed “up” versus “down,” thereby adding a developmental perspective on the embodiment of metaphors. Integrating the observation of object-related action thus gives new impetus to research on the embodiment of metaphors linking emotional valence and vertical space.

## Compliance with Ethical Standards

All procedures performed in studies involving human participants were in accordance with the ethical standards of the regional ethics committee and with the 1964 Helsinki declaration and its later amendments or comparable ethical standards. Informed consent was obtained from all individual participants included in the study.

### Conflict of Interest Statement

The authors declare that the research was conducted in the absence of any commercial or financial relationships that could be construed as a potential conflict of interest.
